# Cultural accommodation of internet-based interventions for substance use and related disorders: a proposed comprehensive framework based on a pilot study and a literature review

**DOI:** 10.3389/fpsyg.2023.1063200

**Published:** 2023-06-21

**Authors:** Keren Gueta, Yossi Harel-Fisch, Sophie D. Walsh

**Affiliations:** ^1^Department of Criminology, Bar-Ilan University, Ramat Gan, Israel; ^2^The International Research Program on Adolescent Well-Being and Health, School of Education, Bar-Ilan University, Ramat Gan, Israel

**Keywords:** substance use and related disorders, cultural accommodation, internet-delivered treatment, intersectionality, remote psychotherapy

## Abstract

Despite the low utilization rates of substance use and related disorders services, and the ability of internet-based interventions for substance use and related disorders (IBIS) to address challenges related to service engagement, limited attention has been placed on the processes for the accommodation of these interventions to diverse cultural settings. This study aimed to develop a framework for the cultural accommodation of IBIS across populations based on a pilot study and a literature review. A pilot study of cultural accommodation of an existing internet intervention for alcohol use was carried out in Israel, which involved focus groups and daily online surveys of prospective consumers (*N* = 24) as well as interviews with experts (*N* = 7) in the substance abuse treatment field. Thematic analysis revealed a range of themes that relate to the general Israeli culture and the specific Israeli drinking subculture, identified as needing to be addressed in the process of intervention accommodation. A comprehensive framework for cultural accommodation of IBIS is suggested, consisting of five stages: Technical and cultural feasibility; Engagement of target group; Identification of accommodation variables, Accommodation, and evaluation of the accommodated intervention. In addition, the framework consists of four dimensions of accommodation: Barriers and facilitators; Audio-visual materials and language; Mechanisms of change; Intersectional factors. We suggest that the proposed framework may serve as a guide for the cultural accommodation of existing internet-based interventions for substance use and related disorders across a range of cultural and geographical settings, thus augmenting the ecological validity of internet-based interventions for substance use and related disorders, expanding cross-cultural intervention research, and reducing health disparities worldwide.

## Introduction

Extensive international research has shown that, despite the numerous benefits of treatment centers for substance use and related disorders (SURD, e.g., smoking, problematic alcohol use, and gambling) in Western countries, utilization rates are low. Only one-fifth of those with signs of an SURD sought treatment in 2019 despite its efficacy (Substance Abuse and Mental Health Services Administration, 2020). Worldwide, it has been estimated that fewer than one in six individuals with a SURD problem, receives treatment each year (World Drug Report, 2018).^1^ This treatment gap is due to numerous individual (e.g., shame), social (e.g., fear of stigmatization), structural (e.g., geographical remoteness), organizational (lack of gender sensitive interventions), and economic barriers (e.g., inability to pay for treatment) (Gueta and Addad, 2014; Chebli et al., 2016; Gueta, 2017, 2020). Those barriers are intensified among minorities such as latino adolescents (Burrow-Sanchez et al., 2011), and in geographically remote locations (Arjadi et al., 2015).

Internet-based interventions for substance use and related disorders (IBIS), meaning, the delivery of treatment programs in the form of web sites or mobile applications are well suited to the management of the above barriers given the effectiveness and flexibility associated with internet interventions (Ferreri et al., 2018). In the last decade, varied IBIS has been developed to increase reach, provide real-time monitoring, and offer personalized delivery intervention to tackle a range of SURD (Chebli et al., 2016). IBIS, are part of an umbrella term called E-mental health that encompasses a variety of technological approaches of self-guided interventions to mental health treatment as well as synchronous and asynchronous methods to connect providers to those who need support. Furthermore, the value of IBIS and other digital mental health interventions was recognized during the COVID-19 pandemic for assisting those who do not have access to or do not want traditional face-to-face care (Mark et al., 2022). This issue was particularly relevant to minority groups such as Black, Indigenous, and people of color, since the COVID-19 pandemic intensified the need for culturally responsive mental health services due to higher economic, physical, and mental health effects resulting from the pandemic on those populations (Alvarez et al., 2022). However, despite the need for IBIS during the COVID-19 pandemic, those interventions are less provided by SURD treatment institutions compared to other mental health institutions (Tauscher et al., 2023). This gap is notable since the internet penetration rate, defined as the percentage of the total population who use the Internet has grown globally and can reach up to 67.9% (Internet World Stats, 2022).

Despite the above need and ability of internet-based interventions to reach people around the globe, these interventions are not within reach of many individuals who could benefit from them (Chebli et al., 2016; Muñoz et al., 2018; Salamanca-Sanabria et al., 2018). A systematic review of systematic reviews (Marcolino et al., 2018) which aimed to assess the impact or effectiveness of mobile health interventions in different health conditions, indicated that most studies were performed in high-income countries. The authors concluded, therefore, that there is a necessity to develop and implement computerized interventions in developing countries. For reasons of financial efficiency and validity, this can be facilitated through the adoption of programs that have already been developed elsewhere, for use across cultural and geographical boundaries reducing health disparities worldwide (Gainsbury and Blaszczynski, 2011; Alvarez et al., 2022).

Given the understanding in the cross-cultural psychotherapy literature that culture impacts the content and process of psychotherapy, accommodation of empirically-supported treatment may be best achieved by a standard cultural accommodation (Koç and Kafa, 2019). In this study, cultural accommodation of psychotherapy refers to ‘the systematic modification of an evidence-based treatment or intervention protocol to consider language, culture, and context in such a way that it is compatible with the client’s cultural patterns, meanings, and values’ (Bernal et al., 2009, p.362). Specifically, cultural accommodation of internet-based intervention refers to a systematic, and collaborative process of making changes to a digital health innovation to increase its relevance and acceptability for a local community (Lal et al., 2018). This can increase the acceptability and effectiveness of an intervention for a local population and/or enable the transfer of interventions across cultural and geographical boundaries (Shehadeh et al., 2016; Sundell et al., 2016; Lal et al., 2018; Salamanca-Sanabria et al., 2018). However, little is known about the best methods for cultural accommodation of internet-based intervention given the lack of standardization in the adaptation process in terms of guidelines, procedures, and processes for adaptation as well as the scant documentation of the adaptations made by researchers (Lal et al., 2018; Rathod et al., 2018). This gap is notable in the SURD literature, as the current literature lacks clear guidelines for culturally modifying existing *internet-based interventions*, for substance abuse treatments (Ferreri et al., 2018).

To address this gap in the literature, we sought to develop a comprehensive framework using a mixed emic–etic approach and a multistage procedure (Millward, 2012), synthesizing a literature review (Peters et al., 2015) together with a qualitative pilot study carried out in Israel, on the cultural accommodation of an empirically-supported cognitive-behavioral web-based intervention “Down Your Drink,” (DYD) developed in the U.K for young alcohol-users.

## IBIS: a critical tool in SURD intervention

Across the world, misuse of substances, and problematic behaviors such as gambling remain serious public health concerns (Fowler et al., 2016). Varied IBIS in terms of treatment goals and methods have been developed to tackle these concerns (Chebli et al., 2016). For example, many existing alcohol applications include self-monitoring wherein users are encouraged to regularly monitor their alcohol consumption, while other programs are intended to prevent relapse by incorporating individualized coping strategies (Garnett et al., 2015). Various methods are used, such as multimedia formats, interactive exercises and quizzes, automated tailored feedback, behavioral tools, chat features, and motivational interviewing (Rooke et al., 2013). IBIS varies concerning guidance, as some programs have no interaction between consumer and therapist, and others include regular contact with a therapist through various channels such as email, live chat, or online video-conferencing (Gainsbury and Blaszczynski, 2011), while others combine online peer support group sessions (Cooper, 2004).

Internet-based interventions for substance use and related disorders are available to the general population, for both adults and adolescents, as an alternative to, or as complementing, more formal interventions (Fowler et al., 2016). IBIS have been found to enhance the effects of established treatments among both clinical and subclinical users and to increase the likelihood of users seeking professional help (Fowler et al., 2016). Systematic literature reviews of the effectiveness and treatment outcomes of internet-based interventions for smoking cessation, problematic alcohol use, substance abuse, and gambling, have concluded that internet-based interventions are effective in achieving positive behavioral change (Ferreri et al., 2018; Boumparis et al., 2019; Lin et al., 2019; Kruse et al., 2020; Sagoe et al., 2021). Furthermore, evidence indicates that benefits are associated even with partial program completion (Gainsbury and Blaszczynski, 2011). A recent review of studies also suggests high patient satisfaction with IBIS (Lin et al., 2019; Mark et al., 2022).

### Advantages of IBIS for minority cultures and non-western countries

Internet interventions provide a promising avenue for the widespread and cost-effective delivery of treatment that is accessible, affordable, dependable, individualized, and destigmatized (Fowler et al., 2016). This has particular advantages for minority cultures and members of non-Western countries (Marcolino et al., 2018). IBIS can facilitate access in cases of geographical remoteness, transport problems, physical disabilities, work commitments, or childcare problems that have all been described as barriers to substance use treatment (Gainsbury and Blaszczynski, 2011). The ability of internet interventions to monitor and store electronically user’s client interactions, feedback, drop-out, traffic, and tool utilization data regarding baseline and follow-up data provides a useful platform for evidence-based practice, allowing results across studies to be replicated, extended, and compared with greater ease and clarity (Gainsbury and Blaszczynski, 2011).

Another advantage of IBIS relates to its’ capacity for reducing internal barriers for help-seekers such as stigma, shame, and denial that are intensified among minority populations (Chebli et al., 2016). The perceived anonymity^2^ enabled in IBIS has been found to facilitate self-disclosure, openness, and disinhibition of participants within therapy (Blankers et al., 2012). Studies have also highlighted the benefits of confidentiality (Ferreri et al., 2018), visual appeal, accessibility, interactivity, and the flexibility of treatment mode regarding contact with therapists (Hester et al., 2013), and peer-based social support (Blankers et al., 2012; Kruse et al., 2020).

However, despite the benefits, the therapeutic content offered via IBIS may have limitations when applied to certain racial/ethnic minority clients and across a diverse range of cultural and geographical settings, due to their lack of culturally sensitive accommodation. Several recent meta-analyses show that conventional (i.e., non-internet) forms of treatment effectiveness improve if they are modified or adapted to include cultural variables that relate to the particular cultural needs of various racial/ethnic minorities (Shehadeh et al., 2016; Sundell et al., 2016). For example, a meta-analysis of cultural adaptation of minimally guided interventions for common mental disorders (Shehadeh et al., 2016), indicated that higher cultural adaptation scores were significantly associated with greater effect sizes (*P* = 0.04). Conclusions were the need to create frameworks and to provide information on the methods used to allow comprehensive adaptation to other settings and contexts while keeping fidelity with the original intervention (Shehadeh et al., 2016). Yet, despite the evidence regarding the accommodation of common mental disorders interventions, the particular advantages for racial/ethnic minority cultures and non-westerns countries (Marcolino et al., 2018), and the increasing availability of smartphones in low and middle-income countries,^3^ cultural adaptation in the area of IBIS has not been thoroughly addressed.

## Culturally adapted of internet and non-internet-based interventions

The importance of culturally sensitive or competent therapy has been well theorized, and literature suggests a need for taking into account issues of accessibility and modality as well as the incorporation of culturally-specific elements into the therapeutic process (Rogler et al., 1987; Bernal and Sáez-Santiago, 2006). In line with a culturally competent perspective, cultural adaptation models for *non-internet* interventions have been well-acknowledged as some of them point to specific dimensions of face-to-face interventions that should be modified (Resnicow et al., 1999; Helms, 2015), while other frameworks outline the phases of cultural accommodation (Barrera and Castro, 2006; Hwang, 2009). Regarding the dimensions, one of the first and most widely cited models in the psychosocial intervention literature pertaining to (non-internet) cultural adaptation is the ecological validity model (Bernal et al., 1995). This model, which was originally conceptualized for Latino populations, consists of eight dimensions of interventions (language, persons, metaphors, content, concepts, goals, methods, and context) that can serve as a guide for developing culturally sensitive treatments and adapting existing psychotherapies to specific minority groups. Another cited framework is the Cultural Sensitivity Framework (CSF), which Resnicow et al. (2000) suggested. This framework distinguishes between surface structure and deep structure cultural accommodation to intervention which may be implemented with cultural sensitivity. Surface structure changes aim to improving feasibility by matching materials and messages to outwardly visible characteristics of the target population, such as language, expressions, images, or cultural metaphors. In contrast, deep structure changes target the program’s impact on participants taking into consideration the intersection of social, cultural, and historical variables and core cultural values of a certain population that are relevant to the target behavior.

Additionally, other frameworks propose the phases of cultural accommodation which are needed (Barrera and Castro, 2006; Hwang, 2009). For example, The Cultural Adaptation Process model is a three-phase model (i.e., setting the stage, initial adaptation, and adaptation iterations) which includes research to assess the conceptual fit, determine the needs unique to the community, test adaptations in pilot settings, and make changes using feedback (Bernal et al., 2009). In addition, the Formative Method for Adapting Psychotherapy framework highlights a community-based bottom-up approach for culturally adapting psychotherapy by including stakeholders such as mainstream health and mental health care providers (Hwang, 2009).

However, despite the growing understanding of the need for cultural accommodation of psychological treatment, few models exist which offer guidelines for program adaptation of substance abuse treatment (Burrow-Sanchez et al., 2011). Similar to other psychotherapy interventions (Koç and Kafa, 2019), the efficacy of empirically supported treatments for substance abuse disorders has largely been established in randomized clinical trials with predominantly white samples, which has been criticized from theoretical, ethical, and practice-based viewpoints (Burrow-Sánchez et al., 2015). Such interventions, in use with racial/ethnic minority groups, may impose worldviews of the Western dominant society onto vulnerable populations and be ineffective for minority groups. According to Bernal and Adames (2017), clinicians that strive to employ culturally competent practice to match treatment approaches to their clients’ characteristics and needs, may accidentally risk altering effective components of treatment. As such, they suggest cultural adaptation procedures as a way to balance the tension between maintaining fidelity to evidence-best-practice and the need for psychosocial culturally adapted interventions.

Furthermore, there is a need for more information and research attention on the phases as well as the modification of the dimensions involved in cultural accommodation of internet-based interventions, especially since many of the adaptation frameworks that have been developed were originally meant for face-to-face interventions (Sundell et al., 2016; Lal et al., 2018; Alvarez et al., 2022). Specifically, regarding substance abuse treatment, the Cultural Accommodation Model for Substance Abuse Treatment (CAM-SAT) (Burrow-Sanchez et al., 2011) was developed as a framework for guiding the development and testing of culturally accommodated versions of (face-to-face) treatment content and delivery to increase cultural relevance for Latino adolescents. The model includes the possibility of adding additional, culturally specific, modules to the existing program (e.g., on the issue of ethnic identity and adjustment). A recent meta-analysis indicated that culturally sensitive substance use treatments for racial/ethnic minority youth had greater reductions of post-treatment substance use levels (*g* = 0.37) compared to other conditions (Steinka-Fry et al., 2017).

However, the introduction of web–based interventions demands consideration of additional aspects of cultural accommodation since it applies to the needs and expectations of potential users about the digital medium itself and not only the intervention’s content (Burchert et al., 2019). Relying on face-to-face accommodation frameworks may not be valid for the accommodation of internet-based interventions given the characteristics of internet-based interventions (Lal et al., 2018; Alvarez et al., 2022). Many previous cultural adaptations neglected to consider user satisfaction, technical literacy, and educational level (Shehadeh et al., 2016). A recent review identified four distinct types of adaptations for culturally adapted internet-based interventions for mental disorders (Spanhel et al., 2021): alterations to an intervention’s structure (e.g., shortening modules), functionality (e.g., accounting for poor internet access), design and aesthetics (e.g., changing graphics to be culturally relevant), and human guidance (e.g., identifying the optimal level of human guidance). Specifically, Lal et al. (2018) in their eHealth adaptation framework for adolescent psychosis identified items that help evaluate users’ experiences of a Web-based platform, for example, motivation, aesthetics, accessibility, interaction, quality, and credibility of information, and usability. They raise technical issues (e.g., internet accessibility), as well as the need for exploring issues such as motivation for internet use, desired level of interaction, and how they prefer to see their community depicted in the intervention. Another study (Burchert et al., 2019) found that Syrian refugees face challenges utilizing mental health apps due to low technical literacy, inadequate language proficiency, a lack of acceptance, and a lack of trust in the app.

Furthermore, internet-based interventions, compared to face-to-face interventions, rely heavily on user engagement (i.e., how actively people are using the program), because they cannot rely on a client-practitioner relationship to establish compliance and adherence (Burchert et al., 2019). Thus, usability in internet-based interventions has become a significant factor in successful intervention development and cultural accommodation (Alqahtani and Orji, 2020). High dropout rates and erratic usage patterns threaten the statistical power and validity of the results of trials, as well as their safety. Thus, exploring usability features can increase adherence and may have a significant effect on the acceptance and accommodation of online mental health interventions (Burchert et al., 2019; Balci et al., 2022).

In addition, an important part of any face-to-face cultural accommodation relates to the client-practitioner relationship such as addressing cultural similarities and differences between them (Bernal et al., 1995) or using treatment staff from the target group (Resnicow et al., 2000). Furthermore, a crucial therapeutic element that also needs to be addressed in face-to-face cultural accommodation relates to professional biases and ethnocultural transference meaning the client’s unconscious diversion of emotions from someone in his life to the therapist (Hwang, 2009). However, those issues may be non-relevant to IBIS as some of the online interventions are self-guided or include only minimal contact from therapists. Instead, those client-practitioner relationship issues in the internet arena may take other forms or may introduce other issues. Moreover, a recent meta-analysis (Balci et al., 2022) of culturally adapting internet-and mobile-based health promotion interventions indicated a limited impact of culturally adapted interventions. This limited impact is attributed to a lack of detailed phases of the adaption process, the limited surface structure of interventions, and the lack of theory and framework of those cultural accommodations.

Thus, given the limited systematics in the cultural accommodation process of internet-based interventions, including IBIS, and the reliance on face-to-face accommodation that may be not valid, there is a need to develop specific internet intervention accommodation frameworks for IBIS that will include both the phases and the dimension of this process (Shehadeh et al., 2016; Abi Ramia et al., 2018; Ferreri et al., 2018). Yet, we were unable to find any models designed specifically for the cultural accommodation of IBIS. Due to the inherent differences between face-to-face and internet- based contexts this lack is notable. The current study aimed to develop an initial framework for cultural adaptation of IBIS, with the help of a pilot study using a well-validated existing IBIS, Down Your Drink (DYD), an online intervention developed in the U.K which we sought to adapt to the Israeli context. Specifically, we aimed to conduct a bottom-up, community-driven qualitative study with a multi-stakeholder perspective of Israeli prospective consumers and experts, to inform the cultural adaptation of an internet-delivered intervention to explore the feasibility, acceptance, and users’ experience of DYD.

## Cultural adaptation of the down your drink (DYD) intervention in Israel: a pilot study

Down Your Drink is an online problem-drinking intervention, originally developed in the U.K. by Linke et al. (2004). DYD includes three phases that assist participants in increasing motivation for change, and provide support during early phases of desistance and assistance in maintaining changes and avoiding relapse (Linke et al., 2008). The original program was designed for use for 6 weeks in a 1-week entry format and is based on the trans-theoretical model of change, the motivational interview, and cognitive-behavioral therapy. Given this therapeutic orientation, DYD is characterized by an enabling and non-confrontational manner reflected both in the style of writing (tone) in the text and the construction of tools aimed at encouraging self-reliance and individual choice (Linke et al., 2008). DYD was developed by researchers and clinicians and has been well-validated and shown good treatment effectiveness. Over the years, the content and presentation of the site have changed according to feedback, advances in the field of short-term interventions, and literature relating to the requirements of interactive sites (Wallace et al., 2011). The program has already been culturally accommodated in additional countries such as Spain (Caballeria et al., 2021). Given the evidence of DYD’s effectiveness and the accommodation to other countries, we aimed to accommodate DYD for delivery in Israel, yet we found a lack of a guiding framework as to how to effectively conduct such cultural adaptation.

### The Israeli social-cultural context

The need for DYD delivery in Israel is rooted, firstly, in the concerning levels of alcohol use among adolescents and young adults. A survey conducted among 348 16–35-year-olds who visited the general emergency departments in Israel during a week indicated that one fifth of those interviewed were in the habit of consuming more than four units of alcohol per drinking session, indicating a high rate of binge drinking among emergency department patients and a need for intervention (Levinson et al., 2017). Statistics from the Israeli Health Behaviors of School-aged Children 2018–19 study found that 21% of Jewish and 12% of Arab 16–18 year olds reported at least one incidence of heavy episodic drinking (more than 5 units) in the past month (Harel-Fisch et al., 2019).

The Israeli social-cultural context offers a compelling case for the exploration of cultural accommodation of IBIS. On the one hand, some characteristics of Israeli culture have been found to serve as a barrier for accessibility to *face-to-face* SURD treatment. Israeli society has been characterized by its strongly masculine and patriarchal nature, in light of the central role of religion in the daily life and the country’s continuous state of war and compulsory army services for all Jewish and some Arab citizens aged 18 (Levy and Sasson-Levy, 2008). This context has been found to serve as a barrier to engagement in face-to-face substance abuse treatment, due to the desire to conceal treatment-related-vulnerability (Gueta et al., 2019). Israel is also a multicultural society composed of diverse ethnic cultures, some of whom experience mistrust in treatment services, creating barriers to face-to-face substance use treatment services (Gueta, 2017). On the other hand, some Israeli social characteristics increase the likelihood of using IBIS and particularly DYD. Israel is characterized by high use of technology: according to the Central Bureau of Statistics, State of Israel (2019), internet penetration in Israel has reached 90% of the population. In addition, the modern Israeli capitalistic lifestyle that treats people in individualistic terms and emphasizes autonomy and self-reliance may be a facilitator to engagement in self-change processes of reducing substance use (Chen et al., 2020). Interestingly, at the same time, Israeli society is also a family-centered society in which family values can serve as a key motivation to drive Israelis to engage in substance use treatment services (Gueta, 2017).

As such, as clinicians and researchers, we believed that Israel is an excellent turf for IBIS. However, we also saw a need for accommodation to the particular social-cultural context. As a first stage, before embarking on a costly process of cultural accommodation, we undertook a pilot study in Israel which included three stages: (1) a week in which study participants were asked to do daily guided use of the English DYD site or, in the case of a control group, daily reading of online self-help literature; (2) Focus groups conducted with the study participants around the experience of their use of DYD or their reading; (3) Seven interviews with experts in the substance abuse treatment field. The findings from the study, together with previous literature, enabled us to map out the dimensions needed for cultural accommodation in Israel, which we believe to have relevance and benefit in additional cultural contexts.

## Methods

### Research paradigm

This qualitative project used a critical realist paradigm (Maxwell, 2012). Ontologically, critical realism claims that a “real world” exists while acknowledging the mediating power of ideology and social and cultural context in producing these realities (Maxwell, 2012). Accordingly, the issue at stake – the cultural accommodation of DYD to the Israeli population manifests in and is shaped by a complex interplay between personal history and experience, interpersonal connections, material conditions, and interactions with social institutions. As this study aimed to create a framework of cultural accommodation for IBIS with a particular focus on practice, a critical realist approach, that can inform policy and practice, provided the appropriate lens to develop this framework. Methodologically, this paradigm influenced the development of the research question, interviews, and data analysis (Maxwell, 2012). Specifically, this paradigm emphasizes comparing and triangulating sources and data types (Maxwell, 2012). Accordingly, this qualitative exploratory research design, including source triangulation features (Patton, 2002), was utilized to explore the feasibility of DYD cultural accommodation from prospective users’ and experts’ perspectives. This triangulation of sources constituted a highly interpretative methodological framework, allowing pragmatic adjustment to an applied setting such as online intervention for substance abuse (Bjelland et al., 2017) Also, following this paradigm affects how data is evaluated and interviews are done (Maxwell, 2012). For instance, under this approach, the interviewer and the interviewee are viewed as active participants in a relationship of mutual learning.

### Procedure and participants

User participants were recruited through social media. A strategy was developed and employed for the recruitment of a non- clinical sample (Chen et al., 2020). We posted an announcement through social media (e.g., Facebook groups of students and groups aimed at research participation). The bold headline read: “Do you want to reduce your drinking on your own?” followed by some information about the project. Phone conversations were held with potential candidates for preliminary selection, based on four eligibility criteria: participants should be aged 18–65 years old, own a computer, currently reside in Israel, and have a high level of English (so they could use the site). In addition, participants were screened with the Alcohol Use Disorders Identification Test (AUDIT), and those with scores lower than 8 or greater than 19 were excluded because DYD is focused on intervention for those drinking hazardously or harmfully, who are likely to be experiencing short-term consequences of their drinking, yet unlikely to be seeking any treatment for this misuse. As a result of the adverts, 35 respondents were found; their screening resulted in a total of 28 participants that began the study. Four participants dropped out of the study due to time constraints, which did not enable them to complete the tasks. There were no notable differences between these participants to those that remained in the study in terms of drinking levels or other socio-economic demographics (e.g., age, gender, education). The final current study comprised 24 problematic alcohol users, 9 women and 15 men, aged 22–30 (*M* = 25). Almost all of them had higher education, were employed, and were single.

Following the screening process, 24 participants were randomly assigned to one of two group- (1) those using DYD (16 participants) and (2) a group assigned to on-line self-help literature (eight participants). The first group was given detailed daily instructions for structured use of the DYD site, to ensure the use of a wide range of tools and interventions within the site. These instructions were developed by the research team following intensive acquaintance with the site, and together with the collaboration of the DYD developers in the UK (details of these tasks are available from the authors). The participants were requested to spend 45 min a day over 1 week on the site, from a personal computer. Participants were requested to provide feedback through an online survey before and after every daily session, to validate the use and gain feedback on the day’s experience. This survey solicited their comments about each specific module of DYD and allowed us to identify on-line directed feedback on three components: general impressions (likes, dislikes, e.g., “What did you like in the program/unit”); usefulness (“What was most helpful/unhelpful?”) and suggestions for modifications to Israeli context and language (“How can we make the program helpful to Israelis?”).

In parallel, the second group was sent daily readings from on-line self-help sites, on issues that also appear on the DYD site, for example, personal stories of recovering addicts, the medical and psychological impact of alcohol use, alcohol norms, etc. They were asked to spend 45 min each day reading the literature and similarly filled out feedback forms each day. They were not informed of the existence of the DYD group. Since only the DYD users were familiar with the DYD website, this group provided feedback only regarding their experience of daily readings from on-line self-help sites and suggestions for cultural accommodation for the Israeli populations based on thus experience.

Next, three focus groups were conducted for the two groups. All groups were audio-taped, lasted about 1.5 h, and followed a written protocol of open-ended questions. The focus group protocol had three main sections (general impressions of experience, perceived usefulness of the different aspects of the site, and suggestions for modifications to the Israeli context). Each session was held in a research room at the university, led by the first and the third authors, who are qualified clinicians (a clinical psychologist and a clinical criminologist) as well as researchers. To compensate for their time, each participant was offered $214 for participation in the week and focus groups. Finally, interviews with seven experts in the substance use field were carried out by the first and the third authors. Interviews with subject matter experts are acknowledged as a primary method in culturally modifying both face-to-face (Resnicow et al., 2000; Hwang, 2009) and internet-based interventions (Alvarez et al., 2022) for gathering input regarding what a community needs from an intervention. The interview started with a presentation of the DYD intervention and the international context of IBIS and was followed by an interview that solicited their comments about DYD and the cultural accommodation of IBIS. The expert participants were a purposive sample, recruited through acquaintances and personal contacts. These were experts in SURDs (i.e., from outpatient counseling and treatment facilities, and rehabilitation), who have long-standing professional experience with the SURD population ranging between 7 to 21 years. The experts, specialists in both psychotherapy and cultural-sensitive treatment, included 2 high-level specialists in relevant government ministries, 4 directors of substance use programs, and an expert in internet intervention. The authors developed the focus group protocol and the interview guide, by carefully considering existing literature on cultural accommodation of SURD interventions and brainstorming ideas to enrich understanding and practice of the topic. In addition, the interview guide was guided by both researchers’ clinical and academic experiences.

### Data analysis

A coding team of the first and the third authors, both involved in research and treatment in the field of SURD, conducted a reflexive thematic analysis of the data using a combination of inductive and deductive approaches (Braun and Clarke, 2012). This methodology was chosen due to its flexibility and variability in theoretical and analytical scope (Braun and Clarke, 2022). Specifically, we drew on the explicit content of the data as this approach is more rooted in the data and therefore is more congruent with a critical realist perspective. We chose to privilege semantic content over latent content, as our goal was to construct a pragmatic framework of IBIS that could inform practice. The choice to conduct an inductive thematic analysis was informed by the understanding that the analysis remains grounded in the data (Braun and Clarke, 2022). Also, this method of study acknowledges the participants’ distinctive DYD experiences in the end and thus continues our earlier ontological and epistemological viewpoint.

**FIGURE 1:**
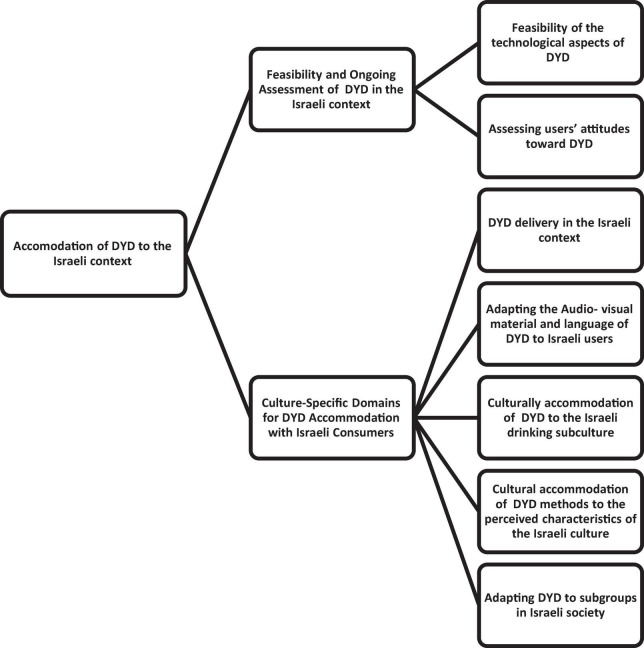
Thematic map of themes and sub-themes.

The themes were actively constructed via the following analytic process. First, using an inductive approach to thematic analysis, the transcribed audio recordings of the focus group and interviews were read and re-read independently by the first and the third authors, to gain greater familiarity with the data and identify initial codes for each participant’s experience of using or perspective of DYD. This was done by writing familiarization notes that reflected the semantic answers and were related to issues such as preferred methods and barriers to using the DYD. Then the research team grouped these notes resulting in systematic data coding. In this phase, codes were created relating to various aspects of the cultural accommodation of the DYD in terms of process and content of the accommodation. Next, the codes s were refined and labeled, and interrelationships between them were proposed resulting in sub-themes according to their topical proximity and conceptual similarity. For example, sub-themes were related to accommodation considering Israeli alcohol use culture (i.e., where drinking occurred, brands of alcohol) or the general Israeli culture (e.g., “Israeli purposefulness”). In the fourth phase, the sub-themes were aggregated under main themes as we developed and reviewed themes. Initially, four preliminary themes were generated, which were then merged and reduced to two themes in the refinement stage. Fifth, our interpretations were theoretically informed, and personal accounts were merged to understand shared meanings (Braun and Clarke, 2019), as we refined and defined the themes. Our reflexive thematic analysis resulted in two related themes, each with several subthemes (see Figure 1). Lastly, “a coherent story about particular patterns of shared meaning across the dataset” (Braun and Clarke, 2019, 592) was developed by the research team. The results which we present involve a synthesis of the themes which came up in the focus groups and the expert interviews together.

### Ethical considerations

The study gained ethical approval from the authors’ university research ethics committee. All participants signed informed consent forms. Following each interview and focus group, the audio recordings were anonymized; a pseudonym was assigned to each participant to protect their identity, and a range of details (such as roles) were deliberately omitted to help protect participant anonymity.

### Reflexivity and quality criteria

Qualitative research, and particularly reflexive thematic analysis, is understood as a subjective process where the researcher brings their “own histories, values, assumptions, perspectives, politics, and mannerisms into the research” (Braun and Clarke, 2013, p. 36). During this study, a thorough audit trail and a reflexive journal were used to critically reflect upon how the authors shaped the research process and impacted theme development (e.g., The third author has personal experience with immigration, having clinical experience working with racial/ethnic minority groups coping with substance abuse problems). Reflection was a continuous process that helped identify and keep track of personal thoughts, feelings, and emotions and shed light on unconscious and changing preconceptions resulting from the emerging study (Sparkes and Smith, 2014). Specifically, we were mindful of our motivations to accommodate the DYD intervention to Israeli society and our values and ethical position regarding the importance of culturally sensitive intervention. Accordingly, we acknowledge the role that our pragmatic position may play in data construction. Thus, the decisions made during data analysis (e.g., reducing the master list of codes) were explicitly outlined to make the study as transparent as possible.

## Results

The analysis of the pilot research participants (both prospective consumers and experts) revealed a high acceptance and perceived efficacy of DYD (see Gueta and Walsh, 2018), but also revealed a wide range of themes identified as targets of DYD accommodation. First, our experience and the pilot study findings indicated a need for ongoing collaboration with the community and stakeholders involved starting with understanding culturally relevant issues relevant to feasibility before starting accommodation and continuing through the process. Second, the participants indicated multiple technical and content recommendations for cultural accommodation of DYD, which interplayed between them. Furthermore, participants indicated that these technical and content issues are shaped by both the general Israeli culture as well as by the specific Israeli drinking subculture.

### Feasibility and ongoing assessment of DYD in the Israeli context

Participants (both prospective consumers but mainly experts) indicated a need for examining technical and cultural feasibility as an essential stage before investing resources in adaptation. In addition, they indicated the need for ongoing evaluation of the accommodation specifically after the implementation. This finding indicated the need for systematic guidelines for the accommodation process, but also for gathering significant knowledge of the community before the accommodation process.

#### Feasibility of the technological aspects of DYD

First, issues that relate to the technological aspects of DYD were suggested by experts for the pre-accommodation process to examine cultural accommodation feasibility to Israeli society and within groups in Israeli society. For example, one treatment expert indicated that the ultraorthodox community in Israel prohibit, based on religious reasoning, the use of smartphones, and limit access to internet sites, thus making IBIS for this community not feasible.

#### Assessing users’ attitudes toward DYD

Second, other within-group relevant cultural variables that the experts identified, related to the need for authorization from a religious figure such as a rabbi or the need for understandings around family relationships and involvement. Specifically, experts highlighted the need to address the target group’s hypersensitivity to labeling, and fear of the criminal justice system. For example, one of the experts advised: “if this program were to be adapted to Israeli Ethiopian immigrants, you should pay attention to the issue of distrust of the formal authorities, such as the police.” Similarly, the consumer participants described a general feeling of mistrust of the “system” (formal institutions such as the police, government offices and national insurance) and said that the site was experienced as a stigmatizing object based on the quantity of alcohol consumption. For example, Amir a 25-year-old described: “the site is pretty tagging like you’re alcoholic and I personally do not feel like I have any kind of drinking problem, I don’t drink as much.” However, other participants, such as Galit pointed to the site as a non-persecutive object characterized by a lack of judgment which takes a respectful approach toward the user: “I really liked the part where the (site) would not always accuse me of… like “you with your drinking habits.”” This variety of reactions toward the DYD site may reflect an internal representation of an object on which various characteristics are cast, based on other internalized relationships of the DYD user and may also relate to cultural perspectives or attitudes toward authority.

Furthermore, regarding the issue of the perceived characteristics of the DYD site and the “relationship” with the DYD site, it was possible to identify a component of a face-to-face therapeutic process, but with unique implications for IBIS, which we conceptualized as “transfer to an internet site.” In this transference process, the website’s users personified the website and referred to it with singular and plural nouns. This is how Ilai, for example, described the site: “The site is very polite.” In addition, it seems that a sort of quasi-therapeutic relationship was formed in which the website was seen as an object from which alcohol consumption must be hidden, as is evident from Adina’s description regarding one of the DYD tools, the diary of documenting alcohol consumption: “The diary stopped me (from drinking) sometimes because somewhere I didn’t want to report any more about it. I wouldn’t say I liked it…There were beers that I almost did not report.”

Lastly, experts also indicated the need for ongoing adjustment and involvement of the consumers and stakeholders in the accommodation process to provide usability needs to increase engagement.

### Culture-specific domains for DYD accommodation with Israeli consumers

The participants (both prospective consumers and experts) discussed the need for accommodation of different aspects of the DYD intervention in order to accommodate it to the Israeli context. Analysis of the interviews and focus groups identified five domains of accommodation: DYD delivery, audio-visual material and language, culturally specific references to alcohol use, cultural preference for type of messages and communication of behavioral change, and sub-groups in Israeli society.

#### DYD delivery in the Israeli context

Delivery relates to the way in which participants gain access to the IBIS and the messages they receive about it prior to use. Participants indicated that in order to accommodate DYD to the Israeli context, barriers and facilitators to DYD related to the intervention *delivery*, rather than to the intervention *content* need to be identified and addressed. For example, according to expert participants, a barrier in the Israeli context for DYD may be related to its use only through a website on a computer and not as an application since Israelis have high levels of mobile phone use. In contrast, in order to facilitate the use of DYD by Israeli consumers, it was suggested by one of the consumers to use social media advertising that highlights the self-change elements of the DYD intervention, given the perceived characteristics of “Israeli roughness” and the need for a sense of control. One of the experts added that “you would need to place the site on a server which is acceptable to the community, … not a site which is seen as stigmatized and unacceptable.”

#### Adapting the audio- visual material and language of the DYD to Israeli users

Another sub theme that was identified by the participants relates to audio- visual material and language. First, the participants related to audio-visual material and language that relates to the general Israeli culture. The site’s users pointed to the need to incorporate pictures in the site, in which the protagonists are Israeli. For example, Gia- a 26-year-old described his recommendations for changes to the site in order to fit the Israeli context: “Totally (pictures) of Israelis, Israeli people, like the first grade English booklets.” Furthermore, participants pointed out various perceived characteristics of the Israeli culture that the accommodation of images needs to address. For example, participants believed there was a need for intense and shocking images, given the high level of emotional intensity among Israelis, to induce behavioral change. They attributed the emotional intensity to threats of terror to Israeli society and Israel’s location in the Middle East with violent conflicts, leading to some emotional numbness among Israelis:

We are a little more immune or more indifferent to what we read and receive through the media because of the place where we live. It’s as if 100 kilometers from here could be a terrorist attack, every moment people explode into pieces…. ….So you’re developing some kind of defense mechanism that even if you’re reading some kind of news then yes, it does not bother me (Shahar, 27-year-old).

In addition, the level of the language was another factor to consider. For example, in our pilot study, we identified a sensitivity to the level of the language used by the (English) DYD that may be interpreted as politeness and condescension from an Israeli standpoint. They suggested that cultural accommodation in Israel would require the writing of verbal content in a simple manner, which would be perceived as writing less formally. For example, Uri, a 27-year-old suggested: “If you translate the site into Hebrew according to the language which is written, for Israelis it will seem patronizing. …The UK is very polite, and the site’s language is too polite for Israelis so use simple and friendly language.”

#### Cultural accommodation of DYD to the Israeli drinking subculture

The pilot participants highlighted various elements of DYD which needed to be adapted *based on the Israeli drinking subculture*. Accordingly, the participants pointed out the need to consider substance use related jargon and sayings such as the word “Satla”: to express idiomatic expressions of the state of being under the influence of drugs (e.g., getting high or feeling drunk). In addition, recommendations for accommodation were related to the need to specify the quantities of alcohol and local alcohol brands, as Ben 24-year-old suggested when referring to the DYD task of creating a drinking diary: “The tables should introduce Israeli drinks. I also really wanted to put the names of the beers on the first day. a little dumb but it makes it a little personal because it’s your beer.” Participants also pointed out the differences in the accepted time and the social circumstances of drinking alcohol in Israel, as opposed to those presented on the DYD. For example, a participant wrote in the online survey about the necessary adjustments to the section on the site where drinking is shown in the morning. “In Israel it is not usual to drink in the morning/lunch, as opposed to Europe.” Another participant, Liran, a 25-year-old emphasized in the focus group the need to present the unique circumstances of Israeli drinking culture: “It is necessary to adapt to situations when we drink in Israel. that it is on Friday nights, events (like weddings).”

In addition, the location of drinking alcohol, such as in home settings and less in pubs, was noted in the online survey as another recommended accommodation of the site: “Examples of social drinking in ‘yeshivas’ (Argo-meaning gathering for drinking) and not necessarily in pubs, I think most of the intensive drinking (in Israel) is done in houses.” There may also be unique areas of recreation and enjoyment for a particular community/culture, such as Eilat (the “holiday city of Israel”) where participants say there is large consumption of alcohol. For example, Galit, a 25-year-old described the unique culture of drinking in Eilat: “Eilat is a kind of bubble, get drunk there no matter how old you are, you need to concentrate on areas in Israel or places where they drink.”

Lastly, participants identified the need to accommodate the site to the special circumstances related to Israeli developmental tasks such as the army service in Israel that may impact drinking habits. For example, during vacation/time off from army service, soldiers may drink excessively and dangerously, as Adina 26-year- old described: “Once you go out for the weekend… you will drink non-stop because you see your friends.”

#### *Cultural accommodation of DYD methods to* the perceived characteristics of the Israeli general culture

The participants indicated various methods of DYD aimed to induce change, which needed to be addressed, based on perceived characteristics of the Israeli culture. Participants related to high levels of self-efficacy among Israelis and “Israeli roughness” which require emphasizing elements of self-change in the process, increasing the user’s sense of control and emphasizing positive motivation. One participant indicated this as an advantage of DYD for the Israeli population and suggested to enhance it:

We’re competitive, with a high ego…. It may be that ego or machoism that everyone sits in a bar and drinks. you don’t want anyone to tell you that you are an alcoholic and you have to change it, …something I really liked on the site the part of taking control yourself …do it even stronger (Tzahi, 34- year-old).

“Israeli purposefulness” (i.e., the need to get straight to the point, not to waste time) was another perceived Israeli characteristic that was highlighted by participants as having implications for types of messages and communication accommodation. This feature was considered to be essential in adapting the site and was reflected in proposals by the participants to reduce the text. For example, referring to “cutting down,” the site’s users wrote that the lengthy wording was less suitable for the Israeli population because “Israelis love ‘t’chless’ (getting straight to the point) and not ‘digging’ (providing unnecessary elaboration as in an archeological excavation (.” In addition, according to the site’s users, adapting to the Israeli characteristics of purposefulness refers to the addition of images, videos and much factual information: “I think that there should be less written and more videos” (Maya, 29-year-old).

Another issue that the participants pointed out regarding the characteristics of the general Israeli society characteristic relates to the interpersonal relationships of the Israeli peer group as having unique implications for the perceived efficiency of techniques on the site. The Israeli peer group was presented by many participants both as a source of pressure but also as a positive influence. Accordingly, an idea was proposed to create an anonymous forum for social comparison of drinking:

The aspect of competitiveness will suit the Israeli population very well. Maybe to integrate social networks. do like a certain forum that is anonymous and where you do not need to be identified and where you see the quantities (of alcohol use) of other people (Zohar, 30-years-old).

The study participants also indicated the need to attend to specific motivations for alcohol use and consumption change in the Israeli context. For example, Oria, 27-year-old related to the use of alcohol in Israel as a means of coping with post-trauma: “… many Israelis drink as a means to cope with their post-traumatic stress disorder, from their military service.” As such, the site would need to relate to the role of alcohol as coping with post trauma and also suggest alternatives, such as therapy for coping. Another issue of motivation for alcohol use and consumption change relates to unique structural issues. For example, participants pointed out the need to present the high costs of alcohol consumption in Israel, compared to Europe, as a motive to reduce alcohol consumption, as Sharona-24-year-old noted: “You can add the money section. It’s really one of the big reasons. I think it’s agreed by most Israelis why you should stop because it really wastes money.” The participants also added the need to sharpen the connection between alcohol consumption and driving, which is of great significance in Israel, due to the lack of comprehensive public transportation, as indicated by Sharona:

The entire issue of drinking in Israel, …is driving. And it does not appear at all on the site, as if in England they are traveling by public transport and that’s it. So, you can put it in as well. Because I was often asked what would prevent you from drinking? So, I straight away said driving, because if my girlfriend decides I’m driving, I cannot drink.

In addition, motivation to change addictive behavior may be related to specific Israeli sets of values, as Yael 25-year-old described the importance of family members in motivating her to reduce her alcohol consumption:

I liked (in the site) that there was the part that you can attach people who are close to you and register not only. your habits, but also it shows the effect on other others. So there was the bit you add a circle with a relative and that, so I added my sister and brother, and suddenly it makes me realize that if I drink then why should my little brother not drink?

Lastly, participants also indicated the need to consider that the perceived effect of DYD may also be influenced by Israeli characteristics and thus demand accommodation. For example, the above-mentioned Israeli numbness and need for a high level of emotional intensity to induce change also shape the long-term effect of the intervention (in comparison to the British need for subtlety or more restrained messages). Accordingly, the underlying reasons which participants thought would lead to attrition were attributed to the absence of internalization of the alcohol damage because the alcohol damage was not shocking enough to resonate in consciousness. For example, Limor 26-year-old noted this issue and her recommendation for addressing this:

I felt that it (the site) gave me tools, but in the end, I did not have anything in my head that echoed for me, which prevented me from drinking. I mean, I was looking for such a shock, like in AHA!! videos…, reminding me, “AHA!!, wait, I saw this no way am I going to drink anymore…you should put horror pictures and stories.

#### Adapting DYD to subgroups in Israeli society

The participants indicated the need to consider subpopulations in Israeli society, that are affected differently by the context of religion and stigmatization that may impact DYD acceptability. For example, one of the experts indicated that for some targeted groups in Israeli society, such as religious Muslims, the DYD goal of moderate drinking may not be relevant since there is a religious prohibition for alcohol use, thus he suggested: “if you accommodate the DYD to Israel, you will need a section in Arabic that its’ goal is not to reduce but rather to stop drinking.” In another example, one of the expert participants pointed out the need to address the gender elevated shame among religious girls: “Girls are more stigmatized because of drinking, particularly girls from religious background maybe you can develop gender-sensitive module.”

## Discussion

The pilot study described here took place within a specific cultural context, in Israel. However, while the specifics of the Israeli-related examples may not apply to other contexts, they may be generalized to the level of overall principles and domains, the content of which may vary from culture to culture. Thus, we wish to use our Israeli pilot study as a prototype to extract and identify key principles and domains of IBIS accommodation that go beyond a specific population or culture. In the discussion, informed as well by the literature review, we present these principles and domains as an initial framework. Thus our framework builds on previous face-to-face cultural adaptation (e.g., Bernal et al., 2009), but also contains the particular considerations feature of designing and implementing interventions for IBIS. Furthermore, the framework that we propose for cultural accommodation is comprehensive as it involves two elements: (1) chronological stages involved in cultural accommodation of IBIS; and (2) dimensions of cultural accommodation.

## Comprehensive framework for the cultural adaptation of ibis

### Stages of cultural accommodation of IBIS

From our analysis of the pilot study, we were able to identify five recommended stages for cultural accommodation for IBIS (see Table 1). Specifically, those stages were developed based on the pilot study’s first theme, which stresses the need for a feasibility and ongoing assessment of DYD in the Israeli context. We start by saying that, as the participants and experts pointed out repeatedly, and which was clear from the comments they made, all of these stages need to take place hand in hand with the targeted community and stakeholders. For example, only those who drink know the names of the drinks, the time they drink, and what messages will speak to them. Only members of a targeted community will know what stigmatizes, what language is needed, and whether the medium is relevant to their community. As such, the findings, and our own experience in interviewing the participants (i.e., our awareness of what we did not know) emphasized how any cultural accommodation demands close collaboration with the target community (Murry and Brody, 2004).

**TABLE 1:** Stages of accommodation.

Stage	Aim	Example issues
Technical and cultural feasibility	To find out if using IBIS is feasible in a specific community	Do they have internet access? Is internet use acceptable to the community?
Engagement	To find out whether the community is motivated to use IBIS	Do potential users feel the site is relevant? Would they want to use it?
Identification of accommodation variables	To identify the culturally specific variables which will need to be translated	What needs to be changed in the existing site? e.g., language, audio visual materials, motivational messages, etc.
Accommodation	To make the actual changes	Translation, changing characters, developing new modules
Evaluation	To assess the effectiveness of the accommodated site	Can potential users navigate the site? Is it user friendly? Do they continue use?

From our readings and analysis of the data, we were able to construct a chronological timeline for carrying out accommodation. The first stage of the model, technical and cultural feasibility (acceptance) relates to assessing the appropriateness of specific interventions to the target group, to ensure that both the content and the delivery of the intervention are acceptable to the target group. According to the relevant literature, this stage can increase the likelihood of uptake and ultimately affect its effectiveness (Escoffery et al., 2018; Lal et al., 2018). This stage, which is unique to IBIS in terms of usability includes exploring technical issues such as internet usage since appropriate internet access (e.g., geographical accessibility, adequate speed) must be widely available to access IBIS. For example, as pointed out by the experts, for some of the Israeli population, DYD may not be relevant due to technology prohibition. Thus, we suggest that this stage needs to involve understanding relevant cultural variables such as attitudes toward technology that may contradict the delivery of IBIS.

Given the numerous themes and ideas for accommodation that were identified by the study pilot and based on the literature (Escoffery et al., 2019), we suggest that the second stage, engagement, will involve the target group (users and stakeholders) in assessing levels of motivation for use of IBIS. This stage aims to further examine the feasibility (acceptance) and appropriateness of specific interventions before the investment of resources in accommodation. In line with the literature, (Shehadeh et al., 2016; Sundell et al., 2016) the current results suggested that working in a researcher–community partnership to develop services is vital as it will increase ownership of the intervention by the local setting and improve its sustainability especially if conducted in a collaborative and shared decision-making process (Lal et al., 2018). Specifically, given the enhanced importance of engagement for internet-based interventions, compared to face-to-face interventions, there is a need to study the usability and the users’ experience by exploring how the IBIS is perceived in terms of engagement and trust and other features of what we have termed “transference to site” to establish compliance and adherence. This issue of stigmatization has been mentioned in cultural accommodation of internet based intervention, but given the intensified stigmatization of people with SURD, comparable to people with mental illness (Corrigan et al., 2009), this issue is critical in IBIS accommodation.

In addition, the study’s findings illustrated that despite the high acceptance and perceived efficacy of DYD to Israeli society and Israeli social characteristics (e.g., high internet use) numerous accommodation domains were identified. Thus we suggest that the third stage of IBIS, the identification of accommodation variables, will identify the most relevant cultural variables, that should be considered in accommodation that will be needed to be integrated into an empirically-supported treatment program (Burrow-Sanchez et al., 2011; Escoffery et al., 2018). This stage can include the identification of cultural values, beliefs, or experiences such as Israeli “roughness” or “purposefulness” or the emphasis on self-control. This can be achieved by identifying and soliciting knowledge from relevant community key stakeholders such as users, families and caregivers, local substance abuse treatment providers, and policymakers. Qualitative methods can allow the stakeholders to express their concerns and suggestions directly to the researcher. This stage also involves a review of the current theoretical and empirical literature regarding the target group. The next stage of the model, accommodation involves the actual changes to the original program, along four dimensions which we describe in the section below. Based on existing literature (Bernal et al., 2009; Escoffery et al., 2019), and face-to-face accommodation and echoed by the experts, we suggest that the fifth stage of the model relates to the evaluation of the accommodated IBIS, in terms of efficacy by randomized clinical trials.

## Dimensions of cultural accommodation of IBIS

The four dimensions which we outline below are the actual elements of the IBIS that we suggest demand accommodation which was derived from thematic analysis of the pilot study interviews and linked to existing literature (see Table 2). Specifically, those dimensions were developed based on the pilot study’s second theme, which stresses the need for culture-specific domains for DYD accommodation with Israeli consumers.

**TABLE 2:** Dimensions of cultural accommodation.

Dimension	Aim	Example
1. Barriers and facilitators to IBIS	Find effective ways to get the target user to find and access IBIS.	Using religious figures, community web sites
2. Audio-visual materials	To make the site attractive and relevant to target users, to enable them to identify.	Changing protagonist names, adding new pictures.
Language and metaphors	Identify relevant cultural expressions and sayings, idioms to increase identification	Identify the unique products and time, age, location and circumstance of substance use.
Cultural context of use	Identify the unique products and time, age, location and circumstance of substance use.	Specify the quantities of alcohol and local alcohol brands, adapt examples to involve appropriate locations and times.
3. Mechanisms of change	Identify the unique cultural motivations and functions of substance abuse for this group.	Substance abuse as “self-medication” with post- traumatic stress, substance use as a response to weakening of family bonds.
The goal of intervention	Identification of cultural values to define goals of the intervention.	Goal of intervention redefined to strengthening family bonds, to reduce alcohol use, to find alternative coping strategies.
Methods	Tasks and procedures employed by the intervention to be acceptable to the client’s culture.	Inclusion of social network or social community features; connection to offline therapists
4. Intersectional factors	Identifying increased vulnerability of multiple marginalized, oppressed or racial/ethnic minority groups.	Role-plays and problem-solving in the context of a racist environment.

### Barriers and facilitators to IBIS

This first dimension involves identifying already existing elements of the IBIS, which may be obstacles (barriers) to the use of IBIS, or in contrast, existing or new elements which may be needed to be introduced to facilitate use in the specific community. In other words, what will prevent or enhance community members’ use of IBIS? In our study, these were seen, for example, by the emphasis on mobile phone use (as opposed to computers) or the need to place the IBIS on a platform (e.g., a website) acceptable to the community. The aim of accommodation in this dimension is to find effective ways to get the target population to find and access IBIS. This domain focuses on the process of intervention delivery rather than on intervention content. It can relate to the initial interface with the IBIS, for example, our pilot, in line with a previous study regarding face-to-face interventions (Gueta, 2017) indicated the need to tackle barriers related to the intensified fear of stigmatization and mistrust in treatment services among racial/ethnic minorities (in the current study, Israeli Ethiopian immigrants) to enhance treatment engagement. Addressing this issue in the context of IBIS can be done by providing details on the technological platform of the intervention, the financing agency, linking the intervention to reputable organizations of the targeted group, and details about online confidentiality to increase the credibility and acceptability of programs by the specific group.

Secondly, the results also indicated a need to identify facilitators for IBIS use, such as enhancing familiarity with the IBIS among the target group. The experts in our study suggested a systematic search for media groups and community web pages for promoting programs. Other methods that were mentioned in the literature can include recruiting and training people with lived experience of the problems (e.g., alcohol users) who receive peer-support training to provide support and foster engagement (Lal et al., 2018). Inherent to this process is the need to involve stakeholders to raise awareness of treatment availability (Barrera and Castro, 2006) and to enhance technological literacy. Moreover, in contrast to face-to-face accommodation, delivery considerations such as a preference for programs that run on a smartphone or tablet over ones that need access to a personal computer due to different rates of technology ownership among minorities (e.g., Black, Indigenous, and people of color) is important to address (Alvarez et al., 2022).

### Audio-visual materials, language, and metaphors

Internet-based interventions for substance use and related disorders, compared to face-to-face interventions, depend on the user connecting to and feeling comfortable with how the site looks and the messages the site is giving. Thus, design and aesthetic components are highly important for the cultural accommodation of IBIS since it promotes user-centered solutions that are based on an evaluation of the demands and living conditions of the target group. Findings from the current study indicated the need to change audio-visual materials (e.g., pictures), language, and metaphors, to amplify and enhance change. This demands a knowledge of which images and graphics are needed to make use of the site attractive to members of a particular population. In prior face-to-face accommodations, videos, and personal stories were revised to include names of the target population (Burrow-Sanchez et al., 2011), typical situations and stereotypes for the targeted group with protagonists such as Colombian actors and Latin American college stories (e.g., economic problems) (Salamanca-Sanabria et al., 2018). However, given the heavy reliance on audiovisual material and the absence of verbal cues in IBIS, there is a need to explore the meaning of such accommodation. For example, as suggested by one of the current study experts and echoed in the literature, the use of images of people from the community may be counterproductive since they may perceive targeted interventions as casting an unfavorable light on their community (Resnicow et al., 2000).

Second, in line with the literature on both face-to-face and internet accommodation, the current study indicated the importance of accommodating surface structures (Resnicow et al., 2000) such as the language, metaphors, cultural expressions and sayings, and idioms, with which the service users may personally identify (Bernal et al., 1995) as well as the level of the language. Furthermore, the current findings also stressed the importance of including substance-use-related jargon and idiomatic expressions related to substance use. Another finding indicated identifying the subculture of substance use in the particular population in terms of the unique products (brands), units, time, location, and circumstance of substance use for the target population, age, and context.

Third, participants in the study related to the centrality of social media in Israel young people’s lives and the idea of including “competition” or comparison. In contrast to face-to-face interventions, many IBIS include the ability to monitor substance use and to compare use with those of a large normative peer sample. Such tailored feedback has been shown to outperform traditional, static health information strategies and is more likely to be read, remembered, and viewed as personally relevant (Bennett and Glasgow, 2009). Studies suggest that incorporating social norms information in feedback helps decrease problematic behavior, such as alcohol consumption, given that individuals often differentially underestimate their own and overestimate the behavior of others (Wood and Williams, 2011). Yet, general peer norms may be seen as lacking credibility for cultural minority members who may seek to compare their use with those of peers within their community (Leightley et al., 2018). This issue suggests the need for a culturally adjusted assessment of SURD symptoms (i.e., what is considered problematic use in a particular culture) since large diagnostic systems have to deal with the tension between universality and cultural specificity since they rely on lists of mostly behavioral criteria (e.g., DSM 5) shaped by social norms about what constitutes heavy drinking or loss of control, that differed considerably between and within cultures and will impact on behavior, as well as on the reporting of behavior (Rehm and Room, 2017).

### Mechanisms of change

Mechanisms of change relate to the active ingredients in existing intervention treatments that account for change (Burrow-Sanchez et al., 2011). Those issues relate to deep structure modification that aimed to enhance the efficacy of the intervention for the target group (Resnicow et al., 2000). Echoing face-to-face accommodation, from the participants’ comments, we saw that this involves identifying the unique cultural motivations and the function of substance abuse for the target group. For example, in the current study, the issue of drinking alcohol as self-medication for war and terror trauma was raised by participants. This is in line with findings regarding the prevalence of past-year and past-month alcohol use among Israeli combatant veterans which was more than 2 times higher among Israeli war veterans than among the general population (Feingold et al., 2019). However, identification of motivations can involve both adding elements to the site which address these motivations and also suggesting alternatives to drinking for fulfilling the needs behind the motivation. Cultural motivations also relate to cultural norms around thinking and approaching tasks such as independence versus interdependence in achieving goals (Burrow-Sanchez et al., 2011). For example, the encouraging self-reliant characteristic of the DYD methods (Linke et al., 2008) was identified in the current study as appealing to the Israeli population in line with the cultural importance of self-change (Chen et al., 2020), but may be less appropriate in cultures which encourage greater levels of interdependence or a sense of self in relation.

Another issue of motivation that was identified, relates to the presentation of the health consequences of SURD (e.g., one of the participants said the health messages were not “shocking” enough). This issue may be related to the well-documented habituation pattern among Israeli citizens in response to chronic terrorism threats that were described through emotional numbness and indifference (Cohen-Louck and Saka, 2017). Thus, we suggest that the accommodation of IBIS should also take into consideration the cultural meaning of SURD health consequences as a motivational factor. For example, the time-orientation preference of the local group which may emphasize long-term health consequences or short-term detrimental consequences to encourage individuals to reduce their alcohol consumption (Burrow-Sanchez et al., 2011). There are also unique structural issues such as the one identified in the current study regarding the cost of alcohol in Israel, compared to Europe, that may enhance alcohol reduction and thus were recommended by participants to be incorporated into DYD.

Secondly, mechanisms of change relate to the goals of IBIS that include the cultural values and customs of the targeted group which are considered as deep structure changes (Resnicow et al., 2000). For example, according to current study findings, and in line with research regarding the Latina population (Bernal et al., 1995), and other Israeli studies (Gueta, 2017), the importance of family values can serve as a main motivation to change alcohol consumption. Thus we suggest that this domain may involve the identification of cultural values that can induce change which may take a very different form in cultures and re-frame the goal of the intervention (Bernal et al., 1995).

Third, Bernal et al. (1995) indicate the need for the methods, tasks, and procedures for problem-solving employed by the intervention to be compatible or acceptable to the client’s culture. For example, studies suggest cultural differences in the extent to which members of a particular ethnic group expect and desire connection with professionals or other users of the intervention site (Fu et al., 2013). This issue is more dominant in IBIS since it relies heavily on those therapeutic methods. Our pilot findings, for example, indicated the desire of users to connect with other users and compare the change in alcohol consumption given the perceived Israeli competitiveness. In other cultures, where anonymity may be more sensitive, this may be highly problematic. Thus, in contrast to face-to-face cultural accommodation that there is a more limited option for delivering the intervention in terms of guidance, we suggest that the accommodation of IBIS should also take into consideration whether the treatment program has the flexibility to consider the needs and the amount of guidance given to the user (e.g., minimal, contact on request or no guidance) which may be more suited in cultural contexts. For example, for certain users, “e-helpers” are used to provide structured guidance which covers a review of the previous session, a review of the user’s experience, and providing support in using the program (Carswell et al., 2018). In addition, compared to face-to-face accommodation that pertains to the relationship with the therapist, the current findings indicated that given the self-guided nature of the intervention, this form of accommodation is not relevant. However, as mentioned above, another process that we termed “transference to site,” relating to internalized relations of a particular cultural group with authority and formal institutions, may be relevant and need to be addressed.

Lastly, another finding of the current study regarding cultural preference for the type of messages and communication relates to the preferred amount of text, images, and emotional intensity that is unique to internet-based intervention (Lal et al., 2018). Specifically, the Israeli preference for minimal words and more images, as well as greater intensity, was attributed to the continual state of war and compulsory army services (Levy and Sasson-Levy, 2008).

### Intersectional and vulnerability factors

In line with previous Israeli findings (Gueta, 2017), the current findings indicated that in addition to accommodation of DYD to the Israeli context, there is a need for addressing within-group differences in Israeli society, such as religious affiliation. This is because users of the site may belong to multiple groups, related to ethnic/racial/migrant groups, gender, sexuality, and social economic class may impact the feasibility of IBIS for them. This is in line with recent evidence from a meta-analysis (Riper et al., 2018) evaluating the effectiveness and moderators of internet interventions for adult drinkers which indicates that stronger effects of digital interventions on alcohol intake were moderated by gender, level of education, and age. Thus, we suggest the need to incorporate an intersectionality lens (Collins, 2015; Gueta, 2020) that can shed light on the dynamics of the intersections among problematic substance use, social identities, and different forms of oppression associated with structural contexts, thus elucidating the complexities of help-seeking behavior of SURD treatment (Gueta, 2017). This is in line with the call of Gonzàlez Castro and Garfinkle (2003) regarding face-to-face cultural accommodation to point out other variables that are critical in the development of culturally relevant substance abuse treatments for specific minority groups such as addressing the within-group differences, regarding different beliefs, attitudes, values, and expectations about treatment that may be reflected in two motivational orientations of modernization and traditionalism.

Specifically, according to the current findings, IBIS accommodation also needs to take into consideration gender roles within the culture and the intersection between substance use and gender such as intense shame. This can mean tailoring toward characteristics of the individual (e.g., clothing) to ensure it is suitable for women or men, and even creating four versions of the main character with users selecting the one they prefer (Carswell et al., 2018). However, this modification may carry the risk of losing the intervention’s internal validity and needs further study.

### Limitations

Limitations of the current study include a relatively small sample size within the participant group categories and sampling including mainly educated students (this was mainly due to the need for participants to have good English to use the UK site). However, although most of the examples provided herein relate to this group of people that are limited in terms of age and education, nonetheless, since we have also based our framework on a literature review of face-to-face and internet-based intervention accommodation, we believe that the principles discussed should apply to other racial/ethnic and sociodemographic subpopulations. Yet, larger samples from other geographical regions and cultures may produce different results or additional dimensions, due to regional and cultural differences and should be a focus of future research. Future research should include focus groups of adolescent participants at different points of the IBIS continuum such as before treatment, during treatment, and post-treatment. This will help to further develop the current preliminary framework of accommodation of IBIS.

## Conclusion

Worldwide, as a growing proportion of the population has easy and affordable access to the internet, and given its’ effectiveness, IBIS represents an innovative cultural accommodation model which can be offered across a range of cultural, geographical, and health system contexts (Shehadeh et al., 2016; Ferreri et al., 2018). The results of the pilot study together with a literature review enabled us to develop a preliminary framework of accommodation of IBIS introducing the stages and dimensions with public health and clinical relevance. Specifically, the novelty and heuristic power of the IBIS framework is twofold. First, in contrast to previous face-to-face accommodation, the current framework stresses usability features given the intensified need to enhance engagement and adherence in IBIS and includes specific features that are relevant only to IBIS such as user experience and engagement, aesthetics, and the intensified substance abuse stigma compared to other mental illness. Second, in contrast to the previous framework that delineates either the phases or the dimensions of cultural accommodation, the current framework is useful as an integrated framework that includes both the phases and dimensions that need to consider in the cultural accommodation of IBIS. This comprehensive perspective is significant as the cultural adaptation processes of internet-based interventions are rarely well-defined or detailed resulting in limiting the efficacy of the accommodations (Balci et al., 2022). In addition, this framework includes both surfaces for improving feasibility as well as deep structural changes to enhance the effect of the intervention (Resnicow et al., 2000) thus addressing previous limitations (Balci et al., 2022). This fills an important gap in the literature and addresses policymakers’ and funding bodies’ need for IBIS accommodation. This is important since accommodation increases the client’s self-management and motivation (Gainsbury and Blaszczynski, 2011), ecological validity, as well as the overall external validity of the intervention (Bernal et al., 1995). Cultural accommodation should ultimately culminate in the conduct of randomized treatment outcome studies that will also contribute to the original interventions. Standardization in cultural adaptations will advance cross-cultural psychotherapy research and practice by enabling us to explore the methodical and efficacy aspects of this process (Koç and Kafa, 2019).

## Author contributions

KG was involved in conceptualizing this work, drafting a major part of the manuscript, revising, and agreeing to be accountable for all aspects of the work. SW was involved in conceptualizing this work, drafting some sections, revising, and agreeing to be accountable for all aspects of the work. YH-F was involved in conceptualizing this work and agreeing to be accountable for all aspects of the work. All authors contributed to the article and approved the submitted version.

## Footnotes

1
www.unodc.org/wdr2018

2 In most cases IBIS sites store site user details and not personal identity-related details.

3
http://www.internetworldstats.com/stats.htm
